# Highly Diverse Hepatitis C Strains Detected in Sub‐Saharan Africa Have Unknown Susceptibility to Direct‐Acting Antiviral Treatments

**DOI:** 10.1002/hep.30342

**Published:** 2019-03-22

**Authors:** Chris Davis, George S. Mgomella, Ana da Silva Filipe, Eric H. Frost, Genevieve Giroux, Joseph Hughes, Catherine Hogan, Pontiano Kaleebu, Gershim Asiki, John McLauchlan, Marc Niebel, Ponsiano Ocama, Cristina Pomila, Oliver G. Pybus, Jacques Pépin, Peter Simmonds, Joshua B. Singer, Vattipally B. Sreenu, Clara Wekesa, Elizabeth H. Young, Donald G. Murphy, Manj Sandhu, Emma C. Thomson

**Affiliations:** ^1^ Medical Research Council ‐ University of Glasgow Centre for Virus Research Glasgow United Kingdom; ^2^ Department of Medicine ‐ University of Cambridge Cambridge Cambridgeshire United Kingdom; ^3^ Wellcome Sanger Institute Hinxton Cambridgeshire United Kingdom; ^4^ University of Sherbrooke Sherbrooke Quebec Canada; ^5^ Medical Research Council/Uganda Virus Research Institute & London School of Hygiene and Tropical Medicine Uganda Research Unit Entebbe Uganda; ^6^ Uganda Virus Research Institute Entebbe Uganda; ^7^ Department of Medicine Makerere University College of Health Sciences Kampala Uganda; ^8^ Department of Zoology University of Oxford Oxford United Kingdom; ^9^ Peter Medawar Building for Pathogen Research University of Oxford United Kingdom; ^10^ National Institute of Public Health of Quebec, Laboratory of Public Health of Quebec Sainte‐Anne‐de‐Bellevue Quebec Canada

## Abstract

The global plan to eradicate hepatitis C virus (HCV) led by the World Health Organization outlines the use of highly effective direct‐acting antiviral drugs (DAAs) to achieve elimination by 2030. Identifying individuals with active disease and investigation of the breadth of diversity of the virus in sub‐Saharan Africa (SSA) is essential as genotypes in this region (where very few clinical trials have been carried out) are distinct from those found in other parts of the world. We undertook a population‐based, nested case‐control study in Uganda and obtained additional samples from the Democratic Republic of Congo (DRC) to estimate the prevalence of HCV, assess strategies for disease detection using serological and molecular techniques, and characterize genetic diversity of the virus. Using next‐generation and Sanger sequencing, we aimed to identify strains circulating in East and Central Africa. A total of 7,751 Ugandan patients were initially screened for HCV, and 20 PCR‐positive samples were obtained for sequencing. Serological assays were found to vary significantly in specificity for HCV. HCV strains detected in Uganda included genotype (g) 4k, g4p, g4q, and g4s and a newly identified unassigned g7 HCV strain. Two additional unassigned g7 strains were identified in patients originating from DRC (one partial and one full open reading frame sequence). These g4 and g7 strains contain nonstructural (ns) protein 3 and 5A polymorphisms associated with resistance to DAAs in other genotypes. Clinical studies are therefore indicated to investigate treatment response in infected patients. *Conclusion*: Although HCV prevalence and genotypes have been well characterized in patients in well‐resourced countries, clinical trials are urgently required in SSA, where highly diverse g4 and g7 strains circulate.

**Abbreviations**
DAA: direct‐acting antiviral drugs
DRC: Democratic Republic of Congo
F protein: fusion protein
g: genotype
HCV: hepatitis C virus
HLA: human leukocyte antigen
NCBI: National Center for Biotechnology Information
NGS: next‐generation sequencing
NS: nonstructural protein
ORF: open reading frame
p‐distance: pairwise distance
SSA: sub‐Saharan Africa
UTR: untranslated region
WHO: World Health Organization

Hepatitis C virus (HCV) prevalence, genotypes, risk factors, and transmission patterns have only been partially characterized in sub‐Saharan Africa (SSA) but are necessary to inform public health policy for preventive and therapeutic strategies in this region. This is particularly pressing in the context of the World Health Organization (WHO) HCV elimination plan, scheduled for 2030. WHO guidelines recommend the use of direct‐acting antiviral drugs (DAAs) as the first‐line therapy for all.^1^ Although DAAs are cheap to manufacture and generic formulations are being rapidly developed, several barriers may hinder efforts to eradicate the virus. Importantly, only 20% of those infected have been diagnosed,^2^ and of those with known infection, some may be infected with strains that confer resistance to DAAs. A lack of knowledge about strains circulating in SSA could affect treatment outcome.^3^ Although far away from clinical use, the main vaccine candidate in clinical trials is based on genotype (g) 1b HCV.^4^

HCV is a member of the diverse *Hepacivirus* genus that includes viruses that infect humans, rodents, bats, canines, and horses.^5^ To date, seven genotypes of HCV have been identified through phylogenetic analysis, which are further subdivided into 84 subtypes, many of which were identified in high‐income countries (HICs).^6^ Additionally, four sequences recently identified in India appear to fulfill the criteria for g8.^7^ The open reading frames (ORFs) of HCV genotypes differ from each other by at least 30% at the nucleotide level, whereas those of subtypes differ by 10%‐25%.^6^ The genome consists of single‐stranded positive‐sense RNA with 5′ and 3′ untranslated regions (UTRs) and 10 genes that encode structural proteins and nonstructural proteins (NSs) (core, envelope E1 and E2, p7, NS2, NS3, NS4A, NS4B, NS5A, and NS5B). Clinical features of infection with different genotypes are similar, with the consequent risk of cirrhosis and hepatocellular carcinoma, but response to treatment varies by genotype.^8^ Encouragingly, pangenotypic combinations of antiviral drugs have recently been licensed; these have wide‐ranging activity against the HCV subtypes present in HICs but have been less well assessed in the context of strains present in low‐income and middle‐income countries, particularly in SSA.^9^

The distribution of HCV genotypes varies substantially around the world.^3^ g1a, g1b, and g3a have a global distribution, whereas subtypes of g3 and g6 are found predominantly in Southern and South East Asia. g4 HCV is associated with infection in East, Central, and North Africa, where up to 20% of some older populations are infected with the virus through historical iatrogenic transmission.^10^, ^11^ Few clinical trials have been carried out in SSA, where g1, g2, g4, g5, and g7 are present, and very few sequences spanning the NS3, NS5A, and NS5B genes are available for analysis of potential resistance mutations.^12^ Many of these genotypes were sequenced in emigrants from Africa who were diagnosed with HCV in other countries, and it is therefore likely that these represent only a small sample of viral strains from a far larger pool of genetic diversity.^13^, ^14^, ^15^, ^16^ Accurate classification is clinically important because treatment response rates and treatment recommendations vary by genotype.^17^ Understanding the extent of HCV genetic diversity would also aid the development of a vaccine to enhance elimination efforts and allow an increased understanding of recent and historical transmission patterns.

We therefore conducted a large‐scale, population‐based study in Uganda to understand the burden of disease and identify strains circulating in this region. We sequenced samples from Uganda and Democratic Republic of Congo (DRC) that were both HCV antibody and RNA positive and samples that were RNA negative but seropositive using unbiased metagenomic sequencing and targeted PCR to investigate the diversity of HCV in this region.

## Participants and Methods

### Human Participants

Patients were recruited in Uganda, DRC, and Canada. Informed consent in writing was obtained from the patients, and the study protocols conformed to the ethical guidelines of the 1975 Declaration of Helsinki as reflected in *a priori* approval by the appropriate institutional review committee.

### Uganda

**Figure 1:**
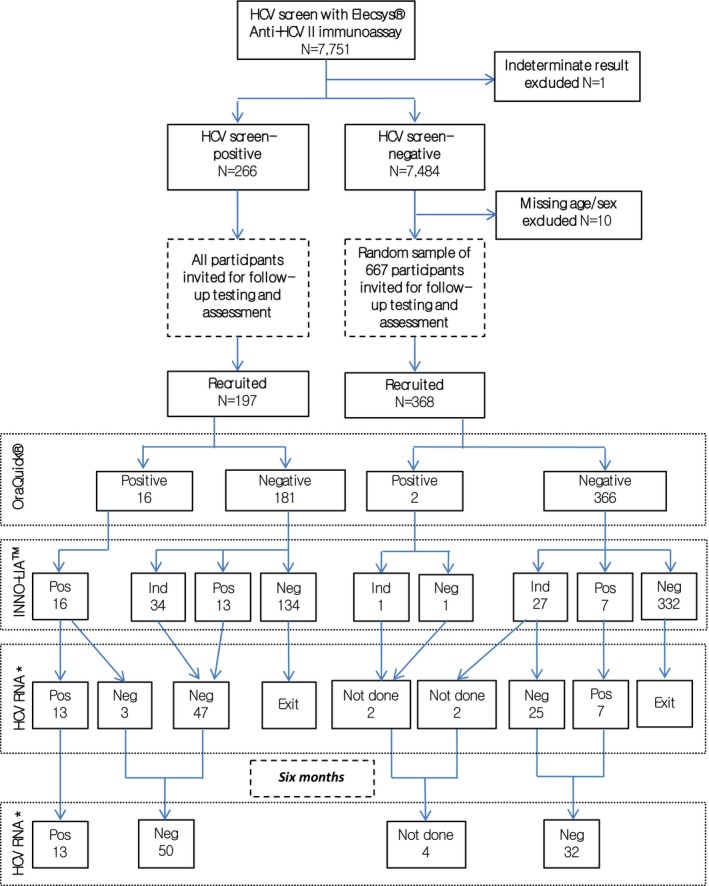
Uganda cohort characteristics. *HCV RNA quantification was carried out using the COBAS®AmpliPrep/COBAS®TaqMan® HCV Qualitative test v 2.0. OraQuick® and INNO‐LIA^TM^ tests were conducted simultaneously.

A cross‐sectional, population‐based survey of participants aged 13 years and older within the Medical Research Council/Uganda Virus Research Institute (MRC/UVRI) General Population Cohort was carried out in 2011,^18^ and individuals were screened for HCV seropositivity. Of 8,056 cohort participants, Elecsys Anti‐HCV II ImmunoAssay screening results were available for 7,751 (Fig. 1). To explore the accuracy of these screening results, all individuals who were seropositive and a randomly selected sample of individuals who were HCV seronegative were invited to participate in a nested case‐control study. Simultaneous baseline testing was carried out with two commercial assays: the US Food and Drug Administration–approved OraQuick HCV Rapid Antibody Test (OraSure Technologies Inc.) and the INNO‐LIA HCV Score Assay (Fujirebio Europe N.V.). Participants with concordant HCV antibody–negative results had no further follow‐up. Participants with antibody‐positive or indeterminate results from either assay underwent quantitative HCV RNA viral load testing, using the COBAS AmpliPrep/COBAS TaqMan HCV version 2.0 (Roche Diagnostics GmbH) at baseline and 6 months later. Statistical analyses were performed using Stata version 12 (StataCorp).

### DRC/Canada

Two patients were identified, both of whom were born in DRC. The first patient (Kin619) was a 70‐year‐old man belonging to the Baluba/Muluba ethnic group, sampled in a survey of elderly patients in Kinshasa^19^ but originating from the Kasai region of DRC. The second sample (QC838) came from a male patient originally from DRC living in Canada. Serology was carried out using an INNO‐LIA assay, and the presence of viral RNA was confirmed by RT‐PCR.

### Biological Samples for Sequencing

A total of 13 × 2 HCV RNA–positive paired samples taken 6 months apart and 11 HCV RNA–negative control samples from the Ugandan study, together with two samples obtained from patients from the DRC, were available for analysis.

### Metagenomic Next‐Generation Sequencing

This was carried out using a metagenomic sequencing protocol as described.^20^ Briefly, RNA was extracted from 200 µL of plasma using the Agencourt RNAdvance Blood Kit (Beckman Coulter) and reverse transcribed using SuperScript III (Invitrogen) with random hexamers and an NEB Second Strand Synthesis Kit (New England Biolabs). Next‐generation sequencing (NGS) using adaptor‐ligation library preparation (KAPA BioSciences) was carried out with several modifications. Complementary DNA was purified with 0.9× AMPureXP magnetic beads (Beckman Coulter) using a “with‐bead” approach. The concentration of DNA was measured with a Qubit 2.0 fluorometer. Adapter‐ligated DNA was amplified in real time on an ABI 7500 cycler, using a KAPA Hifi Real‐Time Library Amplification Kit. Index tags were added using NEBnext multiplex oligonucleotides (New England BioLabs). Amplified DNA was purified using AMPure XP beads and eluted in a final volume of 15 uL. An Agilent 2200 TapeStation was used to verify the final size profile of amplified library DNA. Up to 12 DNA libraries with appropriate index tags were pooled, and 2 × 150–nucleotide paired‐end sequence data sets were generated on an Illumina MiSeq instrument using 300‐cycle v2 reagents.

### Sanger Sequencing

Viral RNA was extracted from 263 µL of serum using the QIAamp Virus BioRobot MDx Kit (Qiagen). Reverse transcription and single‐round DNA amplification by PCR were performed as described.^21^ PCR products were purified and sequenced bidirectionally on an ABI Prism 3100xl genetic analyzer (Applied Biosystems).

### Bioinformatic Analysis

*De novo* assembly (dipSPAdes) and mapping were carried out using Tanoti (http://www.bioinformatics.cvr.ac.uk/tanoti.php). Sequence data were submitted to GenBank (accession numbers to follow). Polymorphisms were identified for currently reported resistance mutations and selected epitopes using coordinates based on the H77 g1a reference strain (AF009606).

Alignments were carried out using MAFFT and manually adjusted as required. Uncorrected pairwise distance (p‐distance) was calculated using MEGA 7.0. Sliding window p‐distance was calculated with a sliding window of 30 base pairs using SSE software. Maximum likelihood phylogenetic analysis was carried out using RaxML (GTR+G+I substitution model). To verify that an HCV strain represented a newly identified genotype, uncorrected genetic p‐distances were calculated between these and the ORF of HCV sequences from all reported strains for which a near‐full genome was available.

Recombination analysis was carried out using the following:

- Bootscanning, which records the statistical support for the grouping of a query sequence within a clade of reference sequences representing each genotype.
- Genetic Algorithm for Recombination Detection (datamonkey.org) using all HCV reference strains.

Patristic distances were calculated using Garli and RAxML. These distances were combined with published estimates of HCV genome evolutionary rates to estimate sequence divergence time. Phylogenetic bootstrap support was calculated using 500‐1,000 bootstrap replicates. HCV reference sequences were obtained from the National Center for Biotechnology Information (NCBI) through the International Committee on Taxonomy of Viruses Web site (https://talk.ictvonline.org/ictv_wikis/flaviviridae/w/sg_flavi/56/hcv-classification) and from the HCV‐GLUE resource (http://hcv.glue.cvr.ac.uk/).

## Results

### HCV Seroprevalence Estimates

In the Uganda cohort, an HCV seroprevalence of 3.4% was estimated using the Elecsys Anti‐HCV II ImmunoAssay (266 HCV participants who were seropositive and 7,484 participants who were seronegative) (Fig. 1). Given the variable performance of HCV antibody screening observed in the Ugandan population^22^ and to clarify the accuracy of screening assays in current use, we carried out a nested case‐control study of 565 participants consisting of 197 cases (74% of screen‐positives) and 368 controls (55% of a random sample of 667 individuals who were seronegative), to undergo additional testing using the OraQuick HCV Rapid Antibody Test and the INNO‐LIA HCV Score Assay.

HCV seropositivity differed substantially by the assay used (Supporting Table S1), indicating a variation in performance among tests that was consistent with other reports. Among 565 Ugandan participants, the OraQuick tested positive in 18 individuals (3.2%), whereas the INNO‐LIA assay tested 36 samples (6.4%) as seropositive. Sixteen individuals had concordant positive serological results.

### Quantitative HCV RNA Viral Load Testing

**Figure 2:**
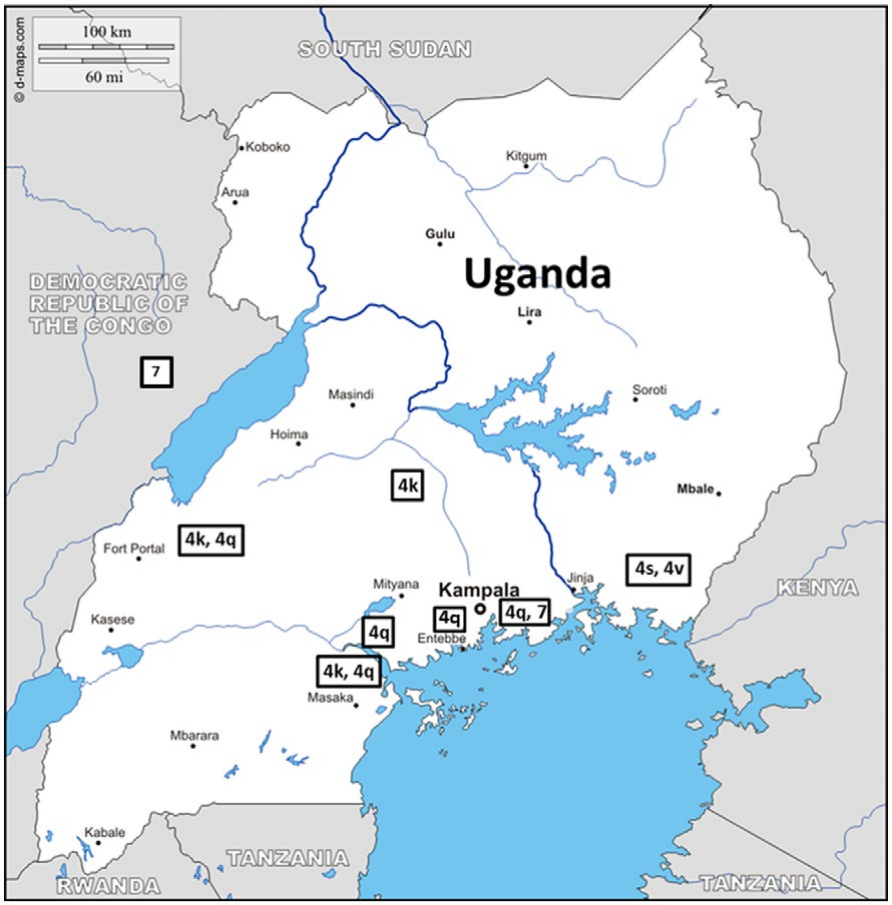
Geographic origin and demographics of HCV infected individuals shown by genotye.

Ninety‐nine (17.5%) of 565 participants had positive or indeterminate results based on OraQuick and INNO‐LIA. Of these, 95 were tested for viral RNA; 20 had detectable viral RNA at baseline (Fig. 2). Six months later, 13/20 (65%) still had detectable RNA indicating chronic infection, whereas 7/20 (35%) had cleared viremia spontaneously.

Using RNA positivity detected by COBAS AmpliPrep/COBAS TaqMan, we compared the utility of the OraQuick and INNO‐LIA for identification of active disease. We found the sensitivity of the OraQuick (65%) was lower than that of the INNO‐LIA (100%). Test “false positives” were more common in the INNO‐LIA assay: Of 75 individuals who were RNA negative, 16 tested positive with INNO‐LIA (specificity 78.7%) compared with 3/75 by OraQuick (specificity 96%). This result may reflect the natural history of spontaneous clearance (or previous treatment) in some individuals or true false‐positive results.

### HCV Sequencing

Genomes containing the full ORF of HCV were obtained for 14/15 participants, and one partial genome sequence was obtained for one patient (QC838). Of these, 12 were identified as g4 and three clustered within g7. Participant characteristics are shown in Table 1. The depth and coverage of each genome obtained using NGS are shown in Supporting Table S2.

**Table 1:** Uganda Cohort Characteristics: Patient Ethnicity, Sex, and Age

Sample ID	Survey	Sex	Age	Ethnic Group
U49	Misenyi	Male	90	Munyarwanda
U100	Kyamulibwa	Male	48	Muganda
U149	Bulwadda	Male	53	Muganda
U150	Bulwadda	Male	65	Muganda
U275	Nakaseeta	Male	48	Munyarwanda
U278	Nalunnya	Female	50	Munyarwanda
U282	Nalunnya	Male	61	Munyarwanda
U288	Nalunnya	Female	77	Murundi
U294	Busoga	Female	67	Muganda
U295	Busoga	Male	60	Munyarwanda
U316	Butiti	Male	67	Munyarwanda
U317	Butiti	Male	69	Munyarwanda
U320	Butiti	Male	60	Mufumbira
Kin619	DRC	Male	70	Muluba
QC838	DRC	Male	—	—

Abbreviation: ID, identifier.

Within g4, four samples were identified as g4k, three were identified as g4v, four were identified as g4q, and one was identified as g4s. Table 2A shows the closest reference genotype of each sample and the p‐distance of each full ORF sequence to the nearest reference genome. Two highly divergent g7 full ORFs were also sequenced (one from the DRC, isolate Kin619‐KP347322, and one from Uganda, isolate U288‐KU861171). The p‐distances of the ORF between these two strains and the nearest reference genome (7a_EF108306) were 30.3% and 20.5%, respectively. These two strains are likely to represent two newly identified subtypes of g7, although confirmation of a new HCV subtype requires the identification of at least two strains for each subtype, according to current classification guidelines^6^ (Table 2B). No evidence of recombination was detected in any sequence.

**Table 2A:** Genotype and Pairwise Distance of Full Coding Region HCV Sequences Compared With the Closest Reference Sequence

Patient	0 Months		6 Months	
	Closest Reference	p‐Distance*	Closest Reference	p‐Distance
U49	4q_FJ462434	0.125		
U100	4k_FJ462438	0.105	4k_FJ462438	0.103
U149	4q_FJ462434	0.107	4q_FJ462434	0.105
U150	4q_FJ462434	0.120	4q_FJ462434	0.120
U275	4k_FJ462438	0.105	4k_FJ462438	0.109
U278	4v_JX227959	0.130	4v_JX227959	0.127
U282	4q_FJ462434	0.179	4q_FJ462434	0.179
U288	7a_EF108306	0.303	7b_KX092342	0.303
U294	4v_JX227959	0.078	4v_JX227959	0.077
U295	4s_JF735136	0.111	4s_JF735136	0.113
U316	4v_JX227959	0.126	4v_JX227959	0.125
U317	4k_EU392171	0.101	4k_EU392171	0.098
U320	4k_EU392171	0.102	4k_EU392171	0.092
Kin619	7a_EF108306	0.205		

* Uncorrected p‐distance.

**Table 2B:** Mean Within and Between Genotype Pairwise Distances of Full HCV Coding Region Reference Sequences

Genotype	Within Genotype	Between Genotype
	p‐Distance*	p‐Distance
1	0.138	0.252
2	0.141	0.261
3	0.157	0.265
4	0.129	0.258
5	0.125	0.265
6	0.174	0.261
7	0.196	—
All	—	0.302

* Uncorrected p‐distance.

Two full g7 ORF sequences were generated using *de novo* assembly from the same Ugandan participant (U288) sampled 6 months apart. Variation across the genome (compared with the H77 reference strain) is shown in Supporting Fig. S1, and divergence over time is shown in Supporting Fig. S2. Over a 6‐month period, four nucleotide substitutions were observed, one of which resulted in an amino acid change within NS4B.

Baseline consensus sequences from the whole g7 ORFs were submitted to GenBank with accession numbers 7*_KU861171 (U288) and 7*_KP347322 (Kin619). The assembled genome sequence of U288 consisted of 9,571 nucleotides, corresponding to nucleotide positions 12 through 9,582 of the H77 reference sequence. The 5′ UTR consisted of 330 nucleotides, and the 3′ UTR consisted of 210 nucleotides. An ORF 3,016 amino acids long was present, and the amino acid lengths of the predicted cleavage proteins were 191 (Core), 192 (E1), 368 (E2), 63 (P7), 217 (NS2), 631 (NS3), 54 (NS4A), 261 (NS4B), 448 (NS5A), and 591 (NS5B). The alternative reading frame gene (F gene), encoding the putative 125 amino acid F protein was also detected (Supporting Fig. S3). The predicted E2 and NS5A proteins of the 7*(U288) sequence contain 1 and 2 additional amino acid differences in length, respectively, when compared with g7a. Genome regions of high variability are evident within the HCV genes E1 and E2, NS4A, NS4B, NS5A, and NS5B. The predicted secondary structure of the 5′ UTR is similar to that of other genotypes of HCV.

For the DRC g7 Kin619 isolate, an ORF of 3,016 amino acids was observed, with amino acid lengths of the predicted cleavage proteins being 191 (Core), 192 (E1), 367 (E2), 63 (P7), 217 (NS2), 631 (NS3), 54 (NS4A), 261 (NS4B), 447 (NS5A), and 592 (NS5B). The putative F protein length was terminated by a stop codon after 11 amino acids.

Using previous low, medium, and high estimates of rate of evolution of g1 HCV (0.000865, 0.001345, and 0.001785 substitutions per site per year respectively)^23^ and an estimate of the patristic p‐distances among the g7 whole‐genome sequences, we estimate that the time of the most recent common ancestor of g7 existed approximately 324 (242‐501) years ago, in the late seventeenth century (calculated by dividing the p‐distance divided by 2 by a previously estimated rate assigned to g1 HCV).

### HCV Phylogenetic Analysis

**Figure 3:**
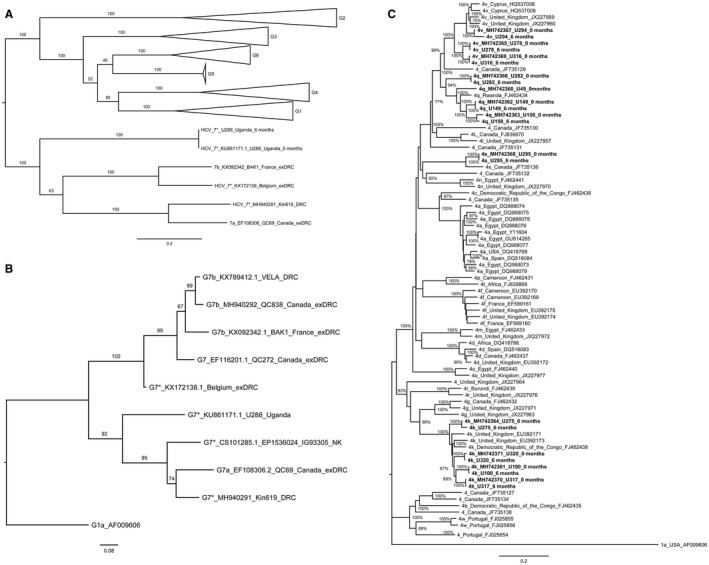
Phylogenetic analysis of HCV sequences from Uganda and DRC. (A) Genotype 7 whole ORF sequences with genotypes 1‐6 (collapsed for ease of viewing). (B) Genotype 7 samples NS5B sequences. (C) Genotype 4 whole ORF sequences HCV genotype was inferred using available whole ORF or NS5B sequences using maximum likelihood, based on the General Time Reversible model with gamma distributed rate among sites with invariant sites (GTR +G+I) and 500 bootstrap replicates. The tree with the highest log likelihood is shown. The H77 genotype 1a strain (AF009606) is included in panels B and C as an outgroup.

Maximum likelihood phylogenetic analysis using available whole and partial reference genomes for g1‐g7 is shown in Fig. 3A‐C and Supporting Fig. S4. Because few whole HCV genomes have been sequenced in SSA (20 available through the HCV‐GLUE resource: accessed on May 21, 2018), a maximum likelihood phylogenetic analysis using only NS5B region sequences was carried out using 65 available g4 strains (Supporting Fig. S4). g4k samples obtained from Uganda were found to cluster closely with g4k strains originating from Rwanda, South Africa, Gabon, the United Kingdom, and Canada. g4q samples were clustered with samples from Burundi and Rwanda; g4v samples were clustered with samples originating in Cyprus, Rwanda, and Burundi; and g4s samples were clustered with a sequence obtained from a Canadian patient originating from East Africa.

The NS5B region phylogenetic analysis also revealed the shared ancestry of the putative newly identified subtypes of g7. The DRC isolate Kin619 clustered most closely with g7a (EF108306), identified in a migrant originating from a similar region within DRC and with short NS5B fragments sequenced from three other DRC nationals living in Canada, DRC, or Belgium (Fig. 3B).^16^, ^24^

### HCV Resistance Analysis

Polymorphisms at sites associated with *in vitro* and *in vivo* resistance in all major genotypes including g7 are shown in Table 3A, and these polymorphisms in the Ugandan g4 samples are shown in Table 3B.

**Table 3A:** Polymorphisms Associated With Resistance: Predicted Resistance‐Associated Variants in HCV Genotype 7

Gene	Location	1a*	1b	2a	3a	4a	5a	6a	7a	7b	7*(U288)	7*(Kin619)
NS3	V36	—	—	L	L	L	L	—	L	L	L	L
NS3	Q41	—	—	—	—	—	—	—	—	—	—	—
NS3	F43	—	—	—	—	—	—	—	—	—	—	—
NS3	T54	—	—	—	—	—	—	—	G	S	—	A
NS3	V55	—	—	—	—	—	L	—	P	—	—	P
NS3	Q80	—	—	G	—	—	K	K	D	E	E	E
NS3	Q86	P	—	P	P	P	P	P	P	P	P	—
NS3	R109	—	—	—	—	—	—	—	—	—	—	—
NS3	R155	—	—	—	—	—	—	—	—	—	—	—
NS3	A156	—	—	—	—	—	—	—	—	—	—	—
NS3	D168	—	—	—	Q	—	E	—	Q	Q	Q	Q
NS3	V170	I	—	I	I	—	—	I	—	R/G	—	—
NS3	E176	—	—	D	S	—	—	—	Q	A	—	Q
NS5A	L23	—	—	—	—	—	—	—	—	—	—	M
NS5A	K24	—	—	T	S	—	Q	Q	—	—	—	—
NS5A	M28	—	—	—	—	V	L	F	L	L	—	L
NS5A	Q30	—	—	—	A	L	—	R	S	S	S	C
NS5A	L31	—	—	M	—	M	—	—	—	—	—	—
NS5A	P32	—	—	—	—	—	—	—	—	—	—	—
NS5A	F37	—	—	—	—	L	—	—	L	L	I	L
NS5A	S38	—	—	—	—	—	—	—	—	—	—	—
NS5A	H54	—	—	T	T	—	S	—	N	T	T	N
NS5A	K56	R	—	R	R	T	—	T	—	M	—	K
NS5A	H58	—	—	P	P	P	P	T	P	P	G	P
NS5A	E62	—	—	N	T	D	T	V	L	N	S	L
NS5A	A92	—	—	C	E	—	—	—	S	—	—	S
NS5A	Y93	—	—	—	—	—	T	T	H	H	H	—
NS5B	L159	—	—	—	—	—	—	—	—	—	—	—
NS5B	S282	—	—	—	—	T	—	—	—	—	—	—
NS5B	C316	—	—	—	—	—	—	—	—	—	—	—
NS5B	L320	—	—	—	—	—	—	—	—	—	—	—
NS5B	V321	—	—	—	—	—	—	—	—	—	—	—

* Reference sequences–accession numbers: 1a: M67463, 1b: EF032892, 2a: AY746460, 3a: X76918, 4a: Y11604, 5a: NC_009826, 6a: AY859526, 7a: EF108306.

**Table 3B:** Polymorphisms Associated With Resistance: Predicted Resistance‐Associated Variants in HCV Genotype 4 Samples From Uganda

Gene	Location	4k	4k	4k	4k	4s	4q	4q	4q	4q	4v	4v	4v
NS3	V36	L	L	L	L	L	L	L	L	L	L	L	L
NS3	Q41	—	—	—	—	—	—	—	—	—	—	—	—
NS3	F43	—	—	—	—	—	—	—	—	—	—	—	—
NS3	T54	—	—	—	—	—	—	—	—	—	—	—	—
NS3	V55	—	—	—	—	—	—	—	—	—	—	—	—
NS3	Q80	—	—	—	—	—	—	—	—	—	—	—	—
NS3	Q86	P	P	P	P	P	P	P	P	P	P	P	P
NS3	R109	—	—	—	—	—	—	—	—	—	—	—	—
NS3	R155	—	—	—	—	—	—	—	—	—	—	—	—
NS3	A156	—	—	—	—	—	—	—	—	—	—	—	—
NS3	D168	—	—	—	—	—	—	E	—	—	—	—	—
NS3	V170	—	—	—	—	—	—	—	—	—	—	—	—
NS5A	L23	—	—	—	—	—	—	—	—	—	—	—	—
NS5A	K24	—	—	—	—	—	—	—	—	—	—	—	—
NS5A	M28	L	L	L	L	—	P	L	L	—	L	L	L
NS5A	Q30	R	R	S	R	—	R	R	R	—	R	R	R
NS5A	L31	—	—	—	—	—	—	M	M	—	M	M	M
NS5A	P32	—	—	—	—	—	—	—	—	—	—	—	—
NS5A	F37	L	L	L	L	—	L	L	L	—	L	L	L
NS5A	S38	—	—	—	—	—	—	—	—	—	—	—	—
NS5A	H54	—	—	—	—	—	—	—	—	—	—	—	—
NS5A	K56	T	T	T	—	T	T	T	T	T	T	R	T
NS5A	H58	P	P	P	P	P	P	P	P	P	P	P	P
NS5A	E62	A	—	—	—	—	—	—	—	—	—	—	—
NS5A	A92	—	—	—	—	—	—	—	—	—	—	—	—
NS5A	Y93	—	—	—	—	—	—	—	—	—	—	—	—
NS5B	L159	—	—	—	—	—	—	—	—	—	—	—	—
NS5B	S282	—	—	—	—	—	—	—	—	—	—	—	—

In the g7 samples, polymorphisms associated with resistance to NS3 and NS5A inhibitors in other genotypes were present in the majority of samples.^25^ Of particular note, the Y93H mutation (associated with a lowered 50% effective concentration [EC_50_] to ombitasvir, daclatasvir, and velpatasvir) is present in the majority of g7 genomes.^26^ The Y93H natural polymorphism is present in only 3% of all sequences submitted to NCBI and occurs at a low frequency in other genotypes (e.g., in HCV g1, it represents 3.16% of sequences submitted to NCBI, and in g3, it represents 4.09% of sequences submitted to NCBI). The D168Q mutation, strongly associated with resistance to first‐generation and second‐generation NS3 protease inhibitors in g3, was also present in all g7 samples.^27^ Although reported as a potential resistance mutation in g1, it is rare in non‐g3 sequences, occurring in 0% of g1 (0/22,386), g2 (0/443), g4 (0/308), g5 (0/57), and g6 (0/584) sequences longer than 500 base pairs submitted to GenBank.

In the 12 Ugandan g4 samples, 36L and 86P in the NS3 gene and H58P in NS5A were observed in all 12 samples sequenced. These are genotype‐specific variants and are also present in the majority of g4 sequences on GenBank. In addition, the NS5A 28L/P and 30R/S variants were present in 83% of sequences (10/12), but the NS5A_28‐32_ MPRMP motif recently associated with resistance in g4r infection was not detected.^9^

### HCV Epitope Analysis

HCV strains sequenced in this study were compared with four well‐characterized epitopes (https://www.iedb.org/) present in the Adenovirus 6/Modified vaccinia Ankara (Ad6/MVA) g1b vaccine in current clinical trials.^28^ The immunodominant human leukocyte antigen (HLA)‐A02‐restricted NS3 1,073 CVNGVCWTV epitope present in the vaccine strain was variable at sites 1, 2, and 6 in the g4 samples from Uganda and at sites 1, 2, 3, 6, 8, and 9 in the g7 samples (Table 4A, 4B, 4C, 4D). Escape variants have not been well described at this site, and further studies will be required to investigate whether cross‐reactive responses are generated with the vaccine. Variation was also present in all sequenced strains when compared with the HLA‐A02–restricted NS3_1,406_ epitope KLSGLGINAV, specifically, at sites 1, 3, 4, and 7 in g4 strains and at sites 3, 4, 5, 7, and 8 in the g7 samples. Within the HLA‐A01–restricted epitope NS3_1,436_ (ATDALMTGY), a Y to F change was present at position nine in the majority of g4 strains and in one g7 strain; this mutation is known to be an escape variant. Finally, the immunodominant HLA‐B27–restricted epitope NS5B_2,841_ ARMILMTHF was also found to contain escape variants in all g4 samples and in g7a. In g7b, 7*(U288) and 7*(Kin619), 2 variants were found that have not previously been tested in immunological studies.

**Table 4A:** Epitope Variation Within Genotype 4 and 7 Strains: NS3_1,073_

Sample	NS3_1,073_ Peptide Motif (HLA‐A02–Restricted)								
Vaccine (g1b)*	C	V	N	G	V	C	W	T	V
Escape variant	None described								
*Genotype 4 samples*									
U100 (4k)	G	I	—	—	—	M	—	—	—
U275 (4k)	G	I	—	—	—	M	—	—	—
U317 (4k)	G	I	—	—	—	M	—	—	—
U320 (4k)	G	I	—	—	—	M	—	—	—
U149 (4q)	A	—	—	—	—	M	—	—	—
U150 (4q)	A	—	—	—	—	M	—	—	—
U282 (4q)	A	—	—	—	—	M	—	—	—
U49 (4q)	A	—	—	—	—	M	—	—	—
U295 (4s)	A	I	—	—	—	M	—	—	—
U278 (4v)	A	—	—	—	—	M	—	—	—
U294 (4v)	A	—	—	—	—	M	—	—	—
U316 (4v)	A	—	—	—	—	M	—	—	—
*Genotype 7 samples*									
EF108306.2 G7a	N	—	G	—	—	M	—	G	—
KX092342.1 G7b	S	I	S	—	—	—	—	S	—
KU861171.1 G7*(U288)	V	C	—	—	—	—	—	—	—
KP347322 G7*(Kin619)	V	L	S	—	—	—	—	A	P

* Ad6 vaccine based on M58335.1 strain.

**Table 4B:** Epitope Variation Within Genotype 4 and 7 Strains: NS3_1,406_

Sample	NS3_1,406_ Peptide Motif (HLA‐A02–Restricted)									
Vaccine (g1b)	K	L	S	G	L	G	I	N	A	V
Escape variant (g1)*	—	—	V	A	—	—	—	—	—	—
Escape variant (g1)	—	—	V	A	—	—	V	—	—	—
Escape variant (g3)	—	—	R	—	M	—	L	—	—	—
Escape variant (g3)	R	—	R	—	M	—	L	—	—	—
Cross‐reactive strain†	—	—	—	—	—	—	L	—	—	—
Cross‐reactive strain	—	—	—	S	—	—	L	—	—	—
*Genotype 4 samples*										
U100 (4k)	Q	—	—	S	—	—	L	—	—	—
U275 (4k)	Q	—	—	S	—	—	L	—	—	—
U317 (4k)	Q	—	—	S	—	—	L	—	—	—
U320 (4k)	Q	—	—	S	—	—	L	—	—	—
U149 (4q)	H	—	—	A	—	—	L	—	—	—
U150 (4q)	Q	—	—	A	—	—	—	—	—	—
U282 (4q)	Q	—	—	A	—	—	V	—	—	—
U49 (4q)	Q	—	—	S	—	—	L	—	—	—
U295 (4s)	Q	—	—	S	—	—	L	—	—	—
U278 (4v)	Q	—	—	G	—	—	L	—	—	—
U294 (4v)	Q	—	L	G	—	—	—	—	—	—
U316 (4v)	Q	—	—	G	—	—	L	—	—	—
*Genotype 7 samples*										
EF108306.2 G7a	—	—	T	S	—	—	L	T	—	—
KX092342.1 G7b	—	—	R	N	A	—	L	—	—	—
KU861171.1 G7*(U288)	—	—	R	T	M	—	—	—	—	—
KP347322 G7*(Kin619)	—	—	T	S	—	—	V	T	—	—

* Variants described in www.iedb.org.

^†^ Cross‐reactive strains but associated with reduced affinity using peptide dilution assays.

**Table 4C:** Epitope Variation Within Genotype 4 and 7 Strains: NS3_1,436_

	NS3_1,436 _Peptide Motif (HLA‐A01–Restricted)								
Vaccine (g1b)	A	T	D	A	L	M	T	G	Y
Escape variant	—	—	—	—	—	—	—	—	F
*Genotype 4 samples*									
U100 (4k)	—	—	—	—	—	—	—	—	F
U275 (4k)	S	—	—	—	—	—	—	—	F
U317 (4k)	—	—	—	—	—	—	—	—	F
U320 (4k)	—	—	—	—	—	—	—	—	F
U149 (4q)	—	—	—	—	—	—	—	—	—
U150 (4q)	—	—	—	—	—	—	—	—	—
U282 (4q)	—	—	—	—	—	—	—	—	F
U49 (4q)	—	—	—	—	—	—	—	—	F
U295 (4s)	—	—	—	—	—	—	—	—	F
U278 (4v)	—	—	—	—	—	—	—	—	—
U294 (4v)	—	—	—	—	—	—	—	—	F
U316 (4v)	—	—	—	—	—	—	—	—	F
*Genotype 7 samples*									
EF108306.2 G7a	—	—	—	—	—	—	—	—	F
KX092342.1 G7b	—	—	—	—	—	—	—	—	—
KU861171.1 G7*(U288)	—	—	—	—	—	—	—	—	—
KP347322 G7*(Kin619)	—	—	—	—	—	—	—	—	—

**Table 4D:** Epitope Variation Within Genotype 4 and 7 Strains: NS5B_2,841_

	NS5B_2,841 _Peptide Motif (HLA‐B27–Restricted)								
Vaccine (g1b)	A	R	M	I	L	M	T	H	F
Escape strain (g3)	V	—	—	V	M	—	—	—	—
Escape strain (g1)	V	—	—	V	—	L	—	—	—
Escape strain (g1)	V	—	—	V	—	—	—	—	—
Escape strain (g1)	V	—	—	V	—	S	—	—	—
Escape strain (g1)	V	—	—	—	—	L	—	—	—
*Genotype 4 samples*									
U100 (4k)	V	—	—	V	—	—	—	—	—
U275 (4k)	V	—	—	V	—	—	—	—	—
U317 (4k)	V	—	—	V	—	—	—	—	—
U320 (4k)	V	—	—	V	—	—	—	—	—
U149 (4q)	V	—	—	V	—	—	—	—	—
U150 (4q)	V	—	—	V	—	—	—	—	—
U282 (4q)	V	—	—	V	—	—	—	—	—
U49 (4q)	V	—	—	V	—	—	—	—	—
U295 (4s)	V	—	—	V	—	—	—	—	—
U278 (4v)	V	—	—	V	—	—	—	—	—
U294 (4v)	V	—	—	V	—	—	—	—	—
U316 (4v)	V	—	—	V	—	—	—	—	—
*Genotype 7 samples*									
EF108306.2 G7a	V	—	—	V	—	—	—	—	—
KX092342.1 G7b	V	—	T	V	F	—	—	—	—
KU861171.1 G7*U288)	V	—	—	V	F	—	—	—	—
KP347322 G7*(Kin619)	V	—	—	V	F	—	—	—	—

### HCV Antibody–Positive and PCR‐Negative Samples

Eleven samples from Uganda from participants with positive HCV serology (Elecsys positive plus either INNO‐LIA or OraQuick positive) but no evidence of HCV RNA by PCR were selected for metagenomic NGS as controls and to look for the potential presence of highly divergent HCV strains not detectable by standard PCR or antigen‐based techniques. No *Hepacivirus* genomes were detected in the samples by mapping or *de novo* assembly‐based methods.

## Discussion

Elimination of HCV will not be an easy task; at least 70 million people around the world are infected, only 20% are aware of their diagnosis, and the roll‐out of new treatments will require major political and financial intervention.^2^, ^29^ In SSA, approximately 11 million people are infected, the majority with genotypes that have received little or no attention in clinical treatment or vaccine trials, and it is likely that genotypes remain undiscovered. At the time of writing, the majority of HCV sequences obtained from SSA represent short regions of the HCV core or NS5B genes, and coverage of whole genes that encode the targets of DAA therapy is extremely sparse.^12^

In our population‐based study in Uganda, we confirmed 20 cases of active HCV infection from 565 individuals tested with multiple serological assays. We noted a preponderance of older individuals infected with HCV (age 48‐90 years). This has been observed in African studies^10^, ^11^ and may reflect a cohort of individuals highly exposed to HCV in the past. Our assessment of HCV seroprevalence demonstrated variable performance between assays, consistent with other estimates for the region. The INNO‐LIA assay was highly sensitive, with low specificity in keeping with false‐positive results and spontaneous clearance in some individuals. Despite high sensitivity and specificity in European and US populations, we found in this Ugandan population that the HCV‐Elecsys ImmunoAssay lacked specificity and sensitivity, below those reported elsewhere,^30^ in keeping with previous seroprevalence studies in Uganda (Supporting Table S3). This was particularly apparent in participants who had an initial positive or indeterminate PCR result but a negative result 6 months later, indicating spontaneous clearance (in which antibody responses are known to lack sensitivity). Molecular techniques have an important role in improving active infection detection rates in SSA to facilitate elimination, and there was no evidence in this study that these lacked sensitivity. Using whole‐genome metagenomic NGS, we found that the most prevalent HCV genotypes in our sample population in Uganda were g4k, g4q, g4v, and g4s. We also sequenced three g7 samples, two of which represent putative newly identified HCV subtypes. Despite a low level of sampling, g7 appears to be highly diverse, with at least three phylogenetic lineages. There is a need to understand more about the diversity of viruses in DRC, Uganda, and other countries in SSA because of the potential impact of viral genetic diversity on diagnostic assay sensitivity, treatment response rates, and vaccine design.

g4 HCV is estimated to have originated in Central Africa in the early eighteenth century.^31^ In this study, we used metagenomic NGS to obtain the first full ORF HCV sequences from participants in Uganda. The most common genotype detected was g4k, strains of which have been detected in persons across Central Africa (DRC, Republic of the Congo, Central African Republic, Rwanda, Cameroon, and Gabon) and North Africa (Tunisia) and in infected migrants in Europe (United Kingdom, France, and Belgium) and North America (Canada). One Ugandan sequence clustered with the g4s reference sequence (obtained from an unspecified region of East Africa^32^). Three samples (each sequenced on two occasions 6 months apart) clustered within g4v alongside sequences obtained from Rwanda, Burundi, and Cyprus. Four samples clustered within subtype g4q alongside sequences from Rwanda and Burundi. In the participants sampled from Uganda, 7/13 were of Munyarwanda ethnicity; this ethnic group represents only 6% of the Ugandan population and was therefore overrepresented in the study. Interestingly, three of these isolates clustered with reference samples from Rwanda (two g4k samples, U275 and U317, and one g4q sample, U282). Given the participant ages, and the history of migration from Rwanda to Uganda in the 1960s and the 1990s, it is likely that some of these individuals were born and infected in Rwanda.

Despite the widespread distribution of these g4 strains, they have not featured in published clinical trials to date (unlike subtypes g4a and g4d).^33^, ^34^, ^35^, ^36^ The impact of resistance‐associated substitutions (RASs) on DAA susceptibility is known to vary by HCV subtype; for example, in g1 HCV, the NS5A Y93H variant causes high‐level resistance in g1a (~600‐fold increase in EC_50_) compared with g1b (<10‐fold). Concerningly, patients with cirrhosis originating from Somalia with the g4r variant were recently found to have a reduced response to DAA treatment in the National Health Service England Early Access Programme in association with the MPRMP NS5A_28‐32_ amino acid motif that is associated with high‐level resistance *in vitro*.^9^, ^37^ In another meta‐analysis of patients with genotype 4 infection treated with ledipasvir and sofosbuvir, treatment failure occurred in two thirds of patients with subtype g4r and in one patient with g4b. These participants had baseline RASs (28M/V+30R+31M), which remained the dominant sequences posttreatment.^38^ The MPRMP motif was not present in the Ugandan samples, but multiple polymorphisms associated with NS5A resistance in other genotypes were present in all samples and included 28L, 31M, 30R, and 58P. Further clinical trials are required to investigate response to DAA treatment in patients infected with these genotypes, particularly in patients at highest risk of lack of response to therapy, such as those with liver cirrhosis. Encouragingly, treatment with newer combination DAA regimens including velpatasvir appears to be highly effective for g4r (sofosbuvir, velpatasvir ± voxilaprevir).^39^

The first full g7 HCV genome (g7a) was first identified in a Canadian migrant from DRC in 2007.^16^ Another highly divergent g7 strain (g7b) was recently identified in a patient also originally from DRC.^24^ Partial genome sequences from four other DRC nationals residing in Belgium, France, and Canada have also been reported. Our study reveals another two highly divergent near‐full g7 genomes (U288 and Kin619) and one newly identified partial genome (QC838), in participants originating from the DRC and Uganda, increasing the recorded diversity and geographical range of this genotype. The identification of a new HCV subtype requires that the nucleotide sequence differs within the coding region by at least 15%, and different genotypes typically differ from each other by 30% at the nucleotide level.^6^ Maximum likelihood analysis of g7 shows that a distinct monophyletic lineage with strong bootstrap support and variation is present across the genome. The U288 sample from Uganda almost meets the criteria for a newly identified genotype (sitting at the upper end of variation found within genotypes but at the lower end for intergenotypic variation). This is comparable with the distance found between more divergent strains of g3 or g6. It has been assigned here to g7 based on phylogeny (genetic distance alone has been shown to be an inadequate measure for assigning new genotypes). No evidence of recombination was detected in these genomes; HCV recombinant genomes are rare but have been reported, including two recently detected g1/4 variants in patients from Cameroon.^40^ The 5′ UTR of the genome is highly structured and relatively conserved compared with other genotypes. The amino acid lengths of the predicted cleavage proteins are similar to those found in the most closely related g7a subtype, with additional insertions in E2 and NS5A. Highest p‐distances were seen in the envelope genes, NS4A, NS4B, and NS5A. An ORF covering 375 nucleotides (125 amino acids) of the putative F protein gene was also present in U288 but not in Kin619. Based on this expanded analysis of g7 sequences, the origin of g7 HCV appears to be in East or Central Africa. Using previous calculations of rate of evolution of HCV, the common ancestor of g7 is likely to have been in the late seventeenth century.

A major concern is the sensitivity of g7 and other genotypes, especially g4 variants prevalent in SSA, to treatment with DAAs and whether reduced sensitivity will affect elimination plans set out by WHO. g7 sequences harbor D168Q and Y93H mutations; these are associated with high‐level resistance to most NS3 protease inhibitors and NS5A inhibitors, respectively. Other variants are present within NS5A and NS3 that require further characterization through *in vitro* replicon and clinical studies. No guidance exists to date for the treatment of g7 infection. Recombinant Japanese fulminant hepatitis 1 (JFH1)/J6 (g2a/1b) strains expressing g7a NS4A/NS5A have been developed but are associated with poor replication; further development of these may aid testing of DAAs in the future.^26^, ^41^ One patient to date has been successfully treated with a combination of sofosbuvir and velpatasvir for g7 infection (mistyped in the ASTRAL‐1 study as g2).^42^ The newer pangenotypic combination therapies sofosbuvir, voxilaprevir, and velpatasvir and glecaprevir and pibrentasvir are less likely to fail in the presence of mutations such as Y93H in other genotypes, so these may prove to be effective; however, no studies in g7 have yet been carried out. g7 HCV has been shown to be mistyped as g2 in other studies using the TRUGENE assay; this could result in errors in therapeutic decision making, and the adoption of full genome sequencing using more sophisticated technologies such as metagenomic and target enrichment–based NGS may reveal other “rare” genotypes as a cause of treatment failure as treatment is rolled out.^9^, ^20^, ^24^ Unbiased metagenomic NGS sequencing overcomes the need for specific primers for full genome sequencing and may therefore help to identify new strains that would not have been amplified using PCR‐based methodology. It does not appear, however, that diagnosis of HCV is impaired with standard assays.

The presence of divergent HCV strains is also of central importance in the efforts to design an effective HCV vaccine. A vaccine candidate designed to target the cytotoxic T lymphocyte response based on an adenoviral prime and MVA boost backbone with the addition of g1b nonstructural genes is in current clinical trials in the United States.^4^, ^28^ When we examined four well‐characterized epitopes likely to be targeted following vaccination with the Ad6 g1b vaccine, described escape mutations were present in the HLA‐B27–restricted NS5B_2,841_ epitope and the HLA‐A01–restricted NS3_1,436_ epitope in the g4 and the g7 strains, whereas variants that have not previously been investigated were present at the HLA‐A02–restricted NS3_1,073_ and NS3_1,406_ epitope sites. Further work will be required to assess cross‐reactivity using these peptides in vaccinated individuals. It is possible that future iterations of the vaccine will require modification at these and other sites for use in SSA.

In conclusion, the prevalence and genetic diversity of HCV in East and Central Africa is only partially characterized and requires further investigation to evaluate diagnostic assay sensitivity, disease burden, and response to treatment and to enhance vaccine design.
