# Supplementary material for: Highly Diverse Hepatitis C Strains Detected in Sub‐Saharan Africa Have Unknown Susceptibility to Direct‐Acting Antiviral Treatments
Source: Hepatology. 2019 Mar 22;69(4):1426–41. doi: 10.1002/hep.30342 (PMC6492010; doi:10.1002/hep.30342)
Supplement: Supplementary file 1 [file HEP-69-1426-s001.pdf]

Supplementary Figure 1: Variation of genotype 7* at the amino acid and nucleotide level compared with genotype 1a

Pairwise uncorrected distance between genotype 7*(U288) and the H77 reference genome is shown plotted across the HCV genome using a sliding window of 30 amino acids (90 nucleotides). Gaps were excluded from the calculation. Areas of high variability are seen within E1, E2, NS4A, NS4B and NS5A. Comparison with other genotypes is available as supplementary information.

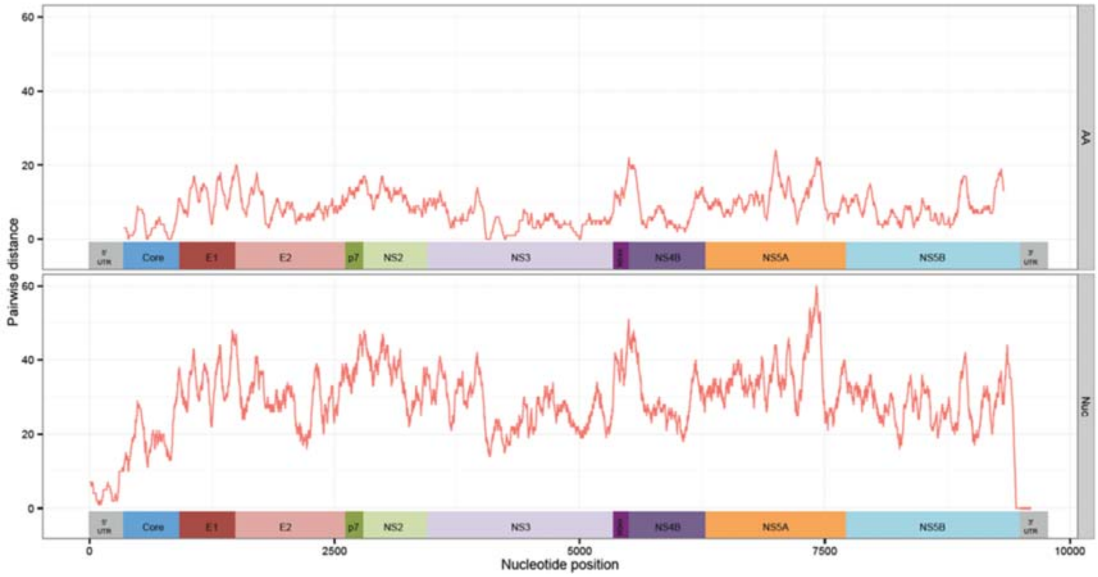

Supplementary Figure 2

Pairwise uncorrected distance between genotype the U288 genotype 7* samples at baseline and 6 months later is shown plotted across the HCV genome using a sliding window of 30 amino acids (90 nucleotides). Gaps were excluded from the calculation.

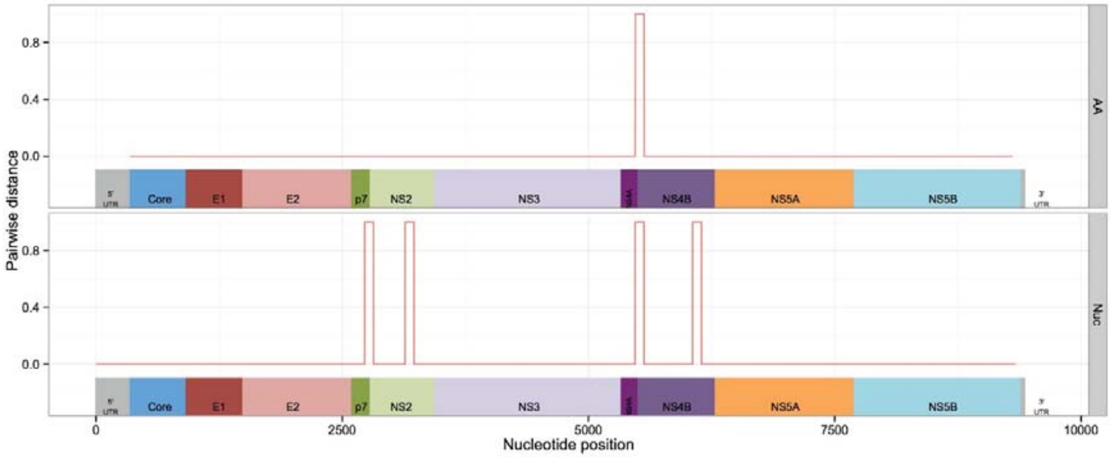

Supplementary Figure 3: The putative F protein gene ORF in selected genotypes

```
                        ....|....| ....|....| ....|....| ....|....| ....|....|
                          10         20         30         40         50

1a_AF00960              MSTNPKPQRK PNVTPTVAHR TSSSRVAVRS LVEFTCCRAG ALDWVCARRG
1b_D90208               MSTNPKPQRK PNVTPTAAHR TLSSRAVVRS LVEFTCCRAG APGWVCARLG
2a_AB04763              MSTNPKPQRK PKETPTVAQK TLSSRAAARS LAEYTCCRAG APGWVCARQG
3a_D17763               MSTLPKPQRK PKETPSVAHR TSSSRVADRS LVEYTCCRAG AHDWVCARRV
4a_DQ41878              MSTNPKPQRK PNVTPTAAPW TLSSRVVARS LAEFTCCRAG APGWVCARLG
5a_AF06449              MSTNPKPQRK PKETPTAAHR TSSSRAVVRS LVEFTCCRAG ALGWVCAQLG
6a_AY85952              MSTLPKPQRK PKETPTVAQW TSSSRVAVRS LAEFTCCRAG APGWVCARRE
7a_EF10830              MSTNPKPQRL PKETPSVAHR TLSSRVAGRS LAESTCCRAG ALDWVCAALG
7*(U288)_KU86117        MSTNPKPQRQ QKETCPVAHR TSSSRAAVRS SAVYTCCLAG AHAWVCAQLG
7*(Kin619)_KP347322     MSTNPKPQRQ P*-------- ---------- ---------- ----------

                        ....|....| ....|....| ....|....| ....|....| ....|....|
                          60         70         80         90         100

1a_AF00960              RLPSGRNLEV DVSLSPRHVG PRAGPGLSPG TLGPSMAMRV AGGRDGSCLP
1b_D90208               RLPSGRNLVE GDNLSPRLAG PRVGPGLSPG TLGPSMATRV WGGQDGSCHP
2a_AB04763              KLRSGPSHVG DASPSPKIGA PLARPGENQV APGPYMGMRD SAGQDGSCPP
3a_D17763               KLLNGHSLAD DDSLSPRRVG AKAGPGLSPG TLGPSMVTRA AGGQGGSCPH
4a_DQ41878              RLRSGRNLVG DANLSPRRVD PREGPGHSQD IHGLFTVMRV VGGRDGSCPP
5a_AF06449              RLQNGRNPVD GVSLSPRRAS PRAGPGVNPG TLGPFMPMRA SGGQGGCSPP
6a_AY85952              RLPSDPSPEA GANLYQRRAS LRAGTGLSPD TLGLFMETRV AGGQVGSCPP
7a_EF10830              RVRSDRSPGE DANVSPKLPL HRVKPGASPG TLGPCMVTRA VAGQGGSCPP
7*(U288)_KU86117        RPPNDHSLGG GASASLRLVP LKGRAGGGLA TPGPFTGMRA AGGQDGSCPP
7*(Kin619)_KP347322     ---------- ---------- ---------- ---------- ----------

                 ....|....| ....|....| ....|....| ....|....| ....|....|
                          110        120        130        140        150
1a_AF00960              VALGLAGAPQ TPGVGRAIWV RSSIPLRAAS PTSWGTYRSS APLLEALPGP
1b_D90208               VALGLVGAPQ TPGVGRVIWV RSSIPLHAAS PTSWGTFRLS APP*------
2a_AB04763              EALAPPGAPL TPGIGRATWV KSSTP*---- ---------- ----------
3a_D17763               AAPVHPGAQM TPGGGPAIWV KSSIP*---- ---------- ----------
4a_DQ41878              VALDRLGAQM IPGGGPAIWV RSSIP*---- ---------- ----------
5a_AF06449              EALGLIGAPM TPGGNRATWV RSSIP*---- ---------- ----------
6a_AY85952              AAPGHIGALM TPGVDPGIWV RLSIP*---- ---------- ----------
7a_EF10830              AALDLLGAP? TPGTARETSV R*STP*---- ---------- ----------
7*(U288)_KU86117        GALALLGAPQ TPGTAPGIWV RSLTP*---- ---------- ----------
7*(Kin619)_KP347322     ---------- ---------- ---------- ---------- ----------


                         ....|....| ....|....| ....|....| ....|
                         160        170        180
1a_AF00960               WRMASGFWKT A*-------- ---------- -----
1b_D90208                ---------- ---------- ---------- -----
2a_AB04763               ---------- ---------- ---------- -----
3a_D17763                ---------- ---------- ---------- -----
4a_DQ41878               ---------- ---------- ---------- -----
5a_AF06449               ---------- ---------- ---------- -----
6a_AY85952               ---------- ---------- ---------- -----
7a_EF10830               ---------- ---------- ---------- -----
7*(U288)_KU86117         ---------- ---------- ---------- -----
7*_(Kin619)_KP347322     ---------- ---------- ---------- -----
```

Supplementary Figure 4: Phylogeny of genotype 4 samples based on partial NS5B sequences and country of origin

Maximum likelihood phylogenetic trees were constructed using RaxML and the GTRGAMMA option with 1000 rapid bootstrap replicates using patientHCV sequences and available NS5B reference sequences labelled with country of sampling and accession number.

S. Fig. 4a) Genotype 4k

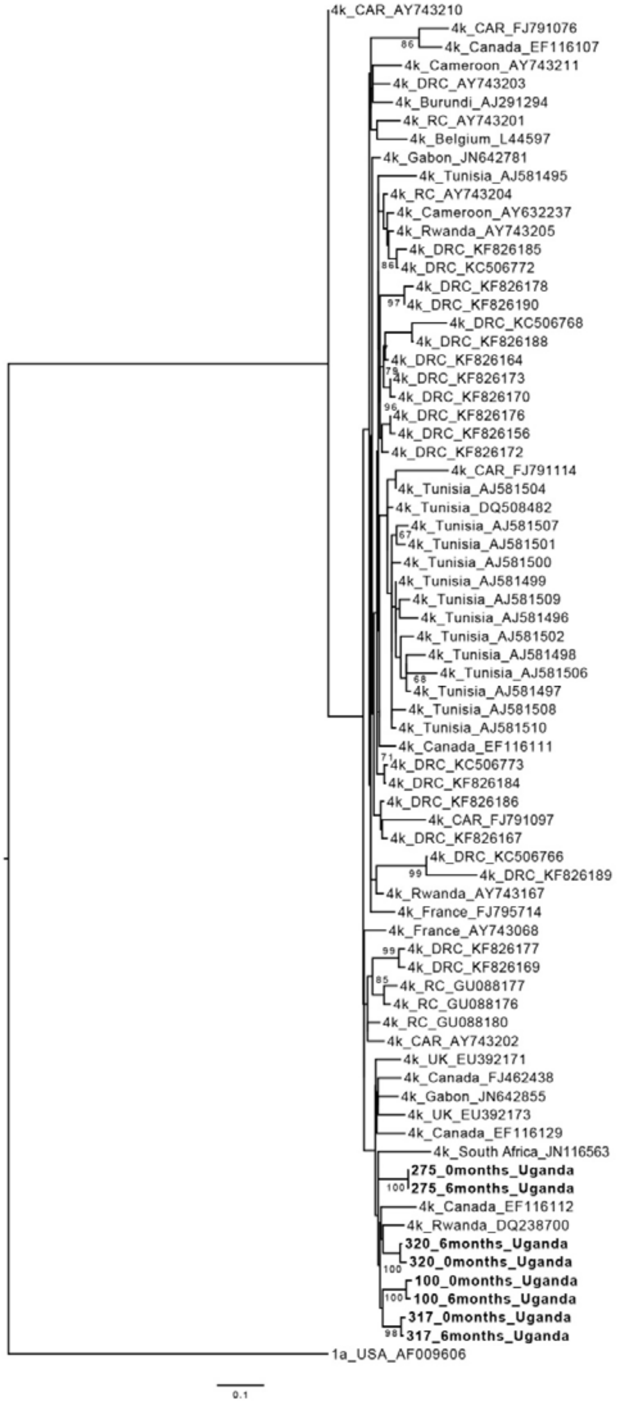

S. Fig. 4b) Genotype 4v

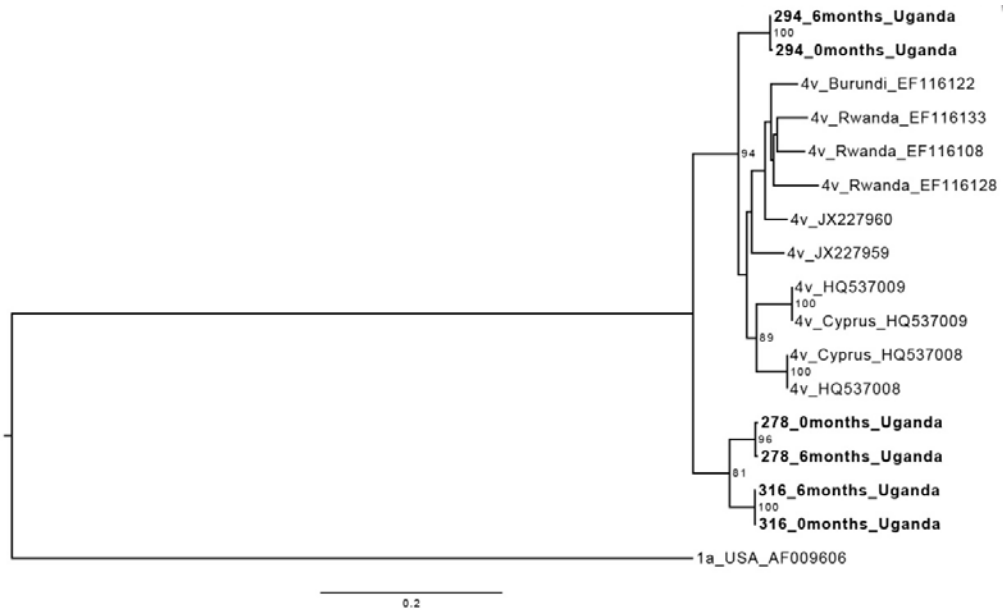

S. Fig. 4c) Genotype 4q

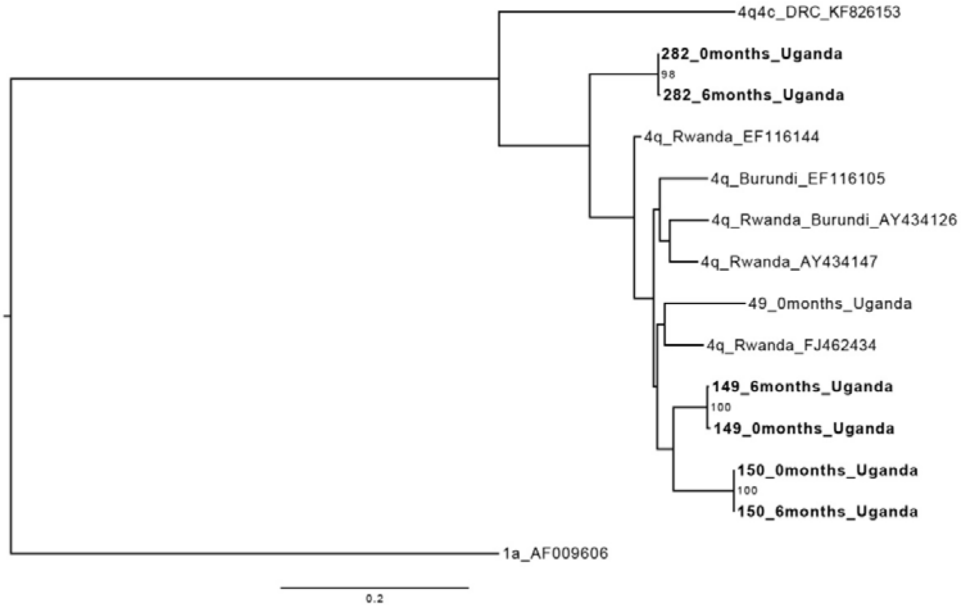

S. Fig. 4d) Genotype 4s

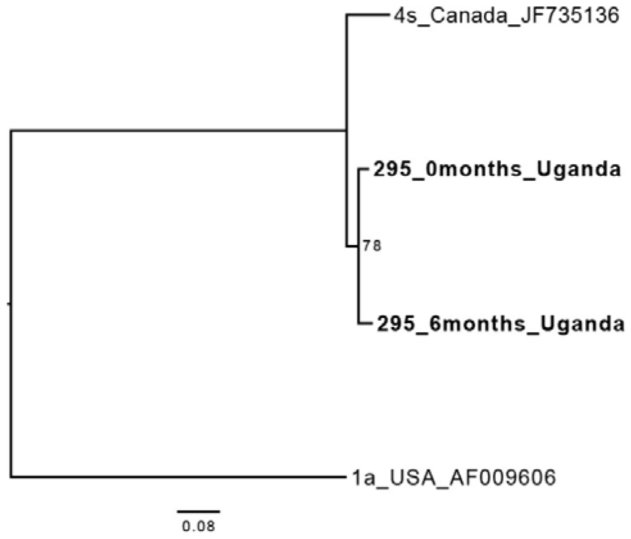

Supplementary Table 1: HCV assay results, by initial screen result (n=565)

		Initial screen result (Elecsys assay)‡			
**Follow-up HCV assay**		**Seropositive N (%)**	**Seronegative N (%)**	**Total N (%)**	**P-value**
**Serum OraQuick® Rapid Test**					
	Positive	16 (8.1)	2 (0.5)	18 (3.2)	**<0.001**
	Negative	181 (91.9)	366 (99.5)	547 (96.8)	
**INNO-LIA™ HCV Score**					
	Positive	29 (14.7)	7 (1.9)	36 (6.4)	**<0.001**
	Negative	134 (68.0)	333 (90.5)	467 (82.7)	
	Indeterminate	34 (17.3)	28 (7.6)	62 (11.0)	
**Concordance between tests (Serum OraQuick® Rapid Test and INNO-LIA™ HCV Score)**					
	Both positive	16 (8.1)	0 (0.00)	16 (2.8)	**<0.001**
	Both negative	134 (68.0)	332 (90.2)	466 (82.5)	
	Discordant	47 (23.9)	36 (9.8)	83 (14.7)	
**Total**		**197**	**368**	**565**	

‡ Elecsys® Anti-HCV II Immunoassay. P-value for Chi-squared test. Not all percentages add up to 100%, due to rounding of numbers

Supplementary Table 2: Depth and coverage of samples sequenced using NGS*

	0months				6months			
Patient	Genome size	Mapped area	Coverage (%)	Average depth	Genome size	Mapped area	Coverage (%)	Average depth
U49	9433	9244	98.00	15				
U100	9418	9373	99.52	5100	8375	8375	100.00	908
U149	9397	9397	100.00	211	9433	9388	99.52	208
U150	9433	9326	98.87	10	9368	9368	100.00	31
U275	9490	9467	99.76	125	9700	7701	79.39	62
U278	9433	9316	98.76	1701	9270	9190	99.14	1072
U282	9700	9686	99.86	1871	9671	9671	100.00	2501
U288	9606	9480	98.69	1235	9589	9589	100.00	3056
U294	9455	9368	99.08	391	9426	8937	94.81	544
U295	9426	9120	96.75	6453	8944	8944	100.00	12567
U316	9281	9275	99.94	15	9549	9549	100.00	524
U317	9281	9281	100.00	1433	9281	9220	99.34	636
U320	9509	9509	100.00	1676	9371	9371	100.00	7560

*Samples from DRC (QC838 and Kin619) were sequenced by Sanger sequencing only

Supplementary Table 3: Previous HCV seroprevalence studies in Uganda

Paper	Assay	Results
**Hladik et al 2005[1]**	ORTHO anti-HCV 3.0 EIA	107/2592 seropositive (4.1%) 15/107 positive by CHIRON recombinant immunoblot assay (RIBA) 3.0 (PCR not done)
**Seremba et al 2010[2]**	ADVIA Centaur HCV assay RSA, Rapidtest® (Cortez Diagnostics, Calabasas, CA)	48/380 seropositive (13%) 14/48 anti-HCV positive patients had detectable serum HCV RNA
**Mullis et al 2013[3]**	Ortho HCV version 3.0 ELISA	7.6% seroprevalence in 1000 individuals tested. All HCV PCR negative.

1. Poor performance of hepatitis C antibody tests in hospital patients in Uganda. Seremba E1, Ocama P, Opio CK, Kagimu M, Thomas DL, Yuan HJ, Attar N, Lee WM. J Med Virol. 2010 Aug;82(8):1371-8. doi: 10.1002/jmv.21817.
2. Hladik W, Kataaha P, Mermin J, Purdy M, Otekat G, Lackritz E, Alter MJ, Downing R. 2006. Prevalence and screening costs of hepatitis C virus among Ugandan blood donors. Trop Med Int Health 11: 951–954.
3. High Frequency of False-Positive Hepatitis C Virus Enzyme-Linked Immunosorbent Assay in Rakai, Uganda Caroline E. Mullis et al Clinical Infectious Diseases, Volume 57, Issue 12, 15 December 2013, 1747–1750
